# Adropin Slightly Modulates Lipolysis, Lipogenesis and Expression of Adipokines but Not Glucose Uptake in Rodent Adipocytes

**DOI:** 10.3390/genes12060914

**Published:** 2021-06-13

**Authors:** Mariami Jasaszwili, Ewa Pruszyńska-Oszmałek, Tatiana Wojciechowicz, Mathias Z. Strowski, Krzysztof W. Nowak, Marek Skrzypski

**Affiliations:** 1 Department of Animal Physiology, Biochemistry and Biostructure, Poznan University of Life Sciences, 60-637 Poznan, Poland; mariami.jasaszwili@up.poznan.pl (M.J.); ewa.pruszynska@up.poznan.pl (E.P.-O.); tatiana.wojciechowicz@up.poznan.pl (T.W.); kwnowak@up.poznan.pl (K.W.N.); 2 Department of Hepatology and Gastroenterology, Charité-University Medicine Berlin, 13353 Berlin, Germany; mathias.strowski@charite.de; 3 Department of Internal Medicine-Gastroenterology & Oncology, Park-Klinik Weissensee, 13086 Berlin, Germany

**Keywords:** adipocytes, adropin, 3T3-L1, lipolysis, lipogenesis, glucose uptake, adipokines

## Abstract

Adropin is a peptide hormone which modulates energy homeostasis and metabolism. In animals with diet-induced obesity, adropin attenuates adiposity and improves lipid and glucose homeostasis. Adropin promotes the proliferation of rodent white preadipocytes and suppresses their differentiation into adipocytes. By contrast, the effects of adropin on mature white adipocytes are unknown. Therefore, we aimed to evaluate the effects of adropin on lipolysis, lipogenesis and glucose uptake in white rodent adipocytes. We assessed the effects of adropin on the mRNA expression of adiponectin, resistin and visfatin. White preadipocytes were isolated from male Wistar rats. Differentiated 3T3-L1 cells were used as a surrogate model of white adipocytes. Lipolysis was measured by the evaluation of glycerol and free fatty acid secretion using colorimetric kits. The effects of adropin on lipogenesis and glucose uptake were measured using radioactive-labelled glucose. The expression of adipokine mRNA was studied using real-time PCR. Our results show that adropin slightly promotes lipolysis in rat adipocytes and 3T3-L1 cells. Adropin suppresses lipogenesis in rat adipocytes without influencing glucose uptake. In addition, adropin stimulates adiponectin mRNA expression and suppresses the expression of resistin and visfatin. These results indicate that adropin may be involved in controlling lipid metabolism and adipokine expression in white rodent adipocytes.

## 1. Introduction

Adropin is a peptide hormone encoded by energy homeostasis-associated (*Enho*), which was originally identified in 2008 by Kumar et al. [1]. The amino acid sequence of secreted adropin contains 43 amino acids and is produced by the proteolytic cleavage of 76 amino acid precursor. The amino acid sequence of adropin is identical to that in rats, mice, humans and pigs [1]. *Enho* mRNA is highly expressed in the brain and liver [1]; however, the presence of *Enho* mRNA and/or adropin peptide was also detected in the kidneys, heart and muscles [2,3]. Notably, adropin is also present in the circulation [4,5,6]. It was found that the biological effects of adropin are conferred through the activation of G protein-coupled receptor 19 (GPR19) [7,8,9]. More than a decade after the identification of adropin, there is convincing evidence demonstrating numerous beneficial effects of this peptide hormone on metabolic diseases such as obesity and diabetes. For instance, it was found that the overproduction or administration of adropin enhances insulin sensitivity, attenuates hepatic steatosis and delays the development of obesity in mice fed a diet enriched in fat [1]. Consistently, adropin deficiency in mice leads to impaired insulin sensitivity, abnormal glucose metabolism and increased adiposity [10]. Furthermore, it was found that adropin may modulate glucose homeostasis by suppressing hepatic glucose production in obese mice [11,12] and by promoting glucose oxidation in skeletal muscle [13]. In addition, several studies showed an inverse correlation between plasma adropin level and body mass index (BMI) [6,14,15,16]. The ability of adropin to protect from increased adiposity in animals fed a high-fat diet and its negative correlation with BMI suggest that adropin may modulate the formation and possibly the functions of adipose tissue. Indeed, recently we reported that adropin promotes the proliferation of rodent white preadipocytes while suppressing their differentiation into mature adipocytes [17]. However, the direct effects of adropin on mature fat cell functions, such as glucose and lipid metabolism, as well as endocrine activity, are largely unknown. Therefore, this study aimed to investigate the effects of adropin on lipolysis, lipogenesis and glucose uptake in white adipocytes. Furthermore, we studied whether adropin is able to modulate the mRNA expression of adiponectin, resistin and visfatin.

## 2. Materials and Methods

### 2.1. Materials

Adropin^34–76^ was synthesized by Novazym (Poznań, Poland). Cell culture media were purchased from Corning B.V. Life Sciences (Amsterdam, The Netherlands). Antibiotics and serum were from Biowest (Nuaillé, France). Total (#PA5-17196), and phospho-HSL (Ser563) (#PA5-104600) antibodies were from Thermo Fisher Scientific (Waltham, MA, USA). Unless otherwise specified, all other reagents were from Sigma-Aldrich (Darmstadt, Germany).

### 2.2. Cell Cultures

Rat primary white preadipocytes were cultured in DMEM/F12 medium supplemented with 10% FBS and a mixture of penicillin (100 kU/L) and streptomycin (100 mg/L). 3T3-L1 cells (a cell model to study adipogenesis and white adipocytes functions [18]) were grown in DMEM containing the same supplements as above. Both cell cultures were maintained in optimal growth conditions (a humidified atmosphere of 5% CO_2_ in air, 37 °C).

### 2.3. Isolation of Rat Preadipocytes

Rat primary white preadipocytes were isolated from epididymal adipose tissue depots of male Wistar rats (body weight 80–100 g, age 5–6 weeks). The fat pads were collected and pooled in a tube prefilled with sterile, freshly prepared Krebs-Ringer solution (KRB) (NaCl 118 mM, KCl 4.8 mM, CaCl_2_ 1.3 mM, KH_2_PO_4_ 1.2 mM, MgSO_4_ 1.2 mM, NaHCO_3_ 24.8 mM, 4-(2-hydroxyethyl)-1-pipera-zineethanesulfonic acid 10 mM) supplemented with 5 mM glucose, 3% BSA and antibiotics (100 kU/L penicillin and 100 mg/L streptomycin). Next, blood vessels were precisely removed, and the tissue was washed with Krebs-Ringer solution at sterile conditions. Subsequently, adipose tissue pads were mechanically minced by scissors and weighed to calculate the amount of collagenase type II needed for digestion. The tissue was digested for 60 min in a water bath at 37 °C and manually shaken every 15 min. Following the collagenase digestion, cells were centrifuged for 10 min at 450× *g* at room temperature (RT). The fat and infranatant were discarded, and the pellet was resuspended in Red Blood Cell Lysing Buffer (Sigma-Aldrich) to lyse the erythrocytes. A 100 µm nylon mesh was used to filter the cell suspension, and after 10 min, the cell suspension was filtered again using 45 µm mesh. Next, Krebs-Ringer solution was added (5 mL), and the cells were centrifuged (10 min at 450× *g* at RT). Afterwards, the supernatant was discarded, and growth medium (DMEM/F12 with 10% FBS and antibiotics) was added to the pellet. Then, cells were counted, and the addition of 0.4% trypan blue allowed their viability to be assessed. Next, cells were seeded in 6- and 24-well plates (depending on the subsequent specific experiments) and maintained in a humidified incubator (37 °C, 5% CO_2_ in air). The differentiation process of rat primary preadipocytes was initiated after 24 h of incubation (see below).

### 2.4. Preadipocyte Differentiation

To conduct the study, preadipocytes were differentiated into mature adipocytes. The differentiation was initiated on the next day after seeding the primary preadipocytes or two days after reaching a 100% confluency of 3T3-L1 cells. According to various research methods, cells were differentiated on 6-well plates (to analyze gene expression and protein production) or on 24-well plates (to assess lipolysis and lipogenesis). The differentiation medium for primary preadipocytes consisted of DMEM/F12, antibiotics (100 kU/L penicillin and 100 mg/L streptomycin), 850 nmol/L insulin, 2 nmol/L T3 and 10 nmol/L dexamethasone. The medium was replaced with fresh differentiation medium every two days until achieving the maturation process. The differentiation of 3T3-L1 cells was induced by the incubation of cells in the growth medium (DMEM, 10% FBS and antibiotics as described above) supplemented with 1 μmol/L dexamethasone, 500 μmol/L 3-isobutyl-1-methylxanthine \and 1 μmol/L insulin. On the third day of differentiation, medium was removed, cells were washed with PBS and incubated for two days in fresh growth medium supplemented with 1 μmol/L insulin. Afterwards, the medium was replaced with a growth medium for an additional two days. After completing the differentiation process, both cell types were incubated in the presence or absence of adropin (0, 10, 100 nmol/L) for different periods of time (see below) and collected for further analyses. All experiments were performed in a serum-deprived medium containing 0.1% fatty acid-free BSA 7–8 days after the onset of the differentiation process.

### 2.5. Lipolysis

Differentiated adipocytes were incubated with or without adropin (1, 10 or 100 nmol/L) for 3 or 24 h in DMEM/F12 medium. Then, the medium was collected and centrifuged (300× *g*) for 10 min. Thereafter, supernatants were collected and stored at −20 °C. Cells were scraped and lysed in RIPA buffer (Sigma-Aldrich) for 10 min on ice. Lysates were centrifuged (14,000× *g* ) for 10 min. Supernatants were collected and stored at −80 °C.

Glycerol was determined using Free Glycerol Reagent (Sigma-Aldrich). Free fatty acids (FFA) were measured using a NEFA HR (2) Kit (Fujifilm, Tokyo, Japan). Secreted glycerol and FFA were normalized to the total protein level, which was measured by a Pierce BCA Protein Assay Kit (Thermo Fisher Scientific).

### 2.6. Lipogenesis

Lipogenesis was measured by evaluation of D-[14C(U)] glucose (Perkin Elmer, Waltham, MA, USA) incorporation into lipids [19]. In brief, rat preadipocytes seeded and differentiated in 24-well plates were treated with or without adropin (10 or 100 nmol/L) for 3 h in DMEM/F12 medium supplemented with 2% fatty acid-free BSA in the presence of D-[14C(U)] glucose. Then, the medium was removed, and cells were lysed using 0.1% SDS. The lipid fraction was separated from the cells using Dole’s extraction method [20]. Lipid phase was transferred into the scintillation liquid, and β-radiation was measured using a Liquid Scintillation Analyzer Tri-Carb 4810 TR (Perkin Elmer).

### 2.7. Glucose Uptake

Rat preadipocytes were seeded and differentiated in 24-well plates. Next, cells were incubated in a glucose-free KRB buffer containing 0.1% BSA for 30 min. After the incubation, cells were washed with PBS and incubated for 30 min in KRB buffer in the presence or absence of adropin (10 or 100 nmol/L). Next, 18.5 kBq of deoxy-D-glucose, 2-[1-14C] glucose (Perkin Elmer) and 0.1 mmol/L 2-deoxyglucose were added, and cells were incubated for 6 min. Then, ice-cold PBS containing 20 μmol/L of cytochalasin B was added. Then, KRB was aspirated, and cells were washed (3 times) with PBS. The cells were lysed using 0.1% SDS. Lysates were transferred into scintillation liquid and β-radiation was measured using a Liquid Scintillation Analyzer Tri-Carb 4810 TR (Perkin Elmer).

### 2.8. Real-Time PCR

Total RNA was extracted using Extrazol reagent (Blirt, DNA Gdańsk, Poland). cDNA was synthetized using FIREScript RT cDNA Synthesis MIX with Oligo (dT) and Random primers (Solis BioDyne, Tartu, Estonia). cDNA was amplified using EvaGreen qPCR Mix (Solis BioDyne) on QuantStudio 12K Flex (Life Technologies, CA, USA). The sequences of PCR primers are shown in Table 1. Relative mRNA expression levels were calculated using the double delta CT method. The expression of mRNA of tested genes was normalized vs. *Gapdh*.

### 2.9. Western Blot

The Western blot technique was used to detect phosphorylated and total HSL. Cells were differentiated and then incubated with adropin (100 nmol/L) for the indicated time points (0–60 min). Next, cell plates were placed on ice and washed with ice-cold PBS. RIPA buffer (Sigma-Aldrich), with an addition of protease and phosphatase inhibitor cocktails (Roche Diagnostics, Mannheim, Germany), was used to lyse cells and to isolate proteins. Tubes with collected cells, after incubation on ice (10 min), were centrifuged (14.000× *g* at 4 °C for 10 min), and then the supernatants were collected. Furthermore, we determined protein concentration by a BCA Protein Assay Kit (Thermo Fisher Scientific). Every sample containing 30 μg of protein was diluted in a loading buffer and then denatured (95 °C, 5 min). SDS-PAGE (5–12% Tris-HCl gel) was applied to separate proteins based on their molecular weight. Afterwards, proteins were transferred onto polyvinylidene difluoride (PVDF) membranes, which were blocked for 1 h at RT in 5% BSA in TBST (50 mmol/L Tris, 100 mmol/L NaCl, 0.1% Tween 20, pH 7.4). We incubated the membranes with a primary antibody (phosphorylated HSL, overnight, 4 °C) and washed them three times (for 10 min each) with TBST. Next, the membranes were incubated with a secondary antibody for 1.5 h at RT and washed again (as described above). Following the last wash, the signal was detected using chemiluminescence (Immobilon Forte Western HRP substrate, Merck Millipore, MA, USA) and visualized by ChemiDoc MP Imaging System (Bio-Rad Laboratories, Hercules, CA, USA). Then, the membranes were stripped and the process was repeated for the total HSL antibody. The dilution was 1:1000 for primary antibodies and 1:5000 for secondary antibodies.

### 2.10. Statistical Analysis

Data were compared using one-way ANOVA followed by the Bonferroni post hoc. *p* < 0.05 (*) was considered to be statistically significant. All experiments were repeated at least two times.

## 3. Results

### 3.1. Adropin Promotes Lipolysis in Rat Primary Adipocytes

**Figure 1:**
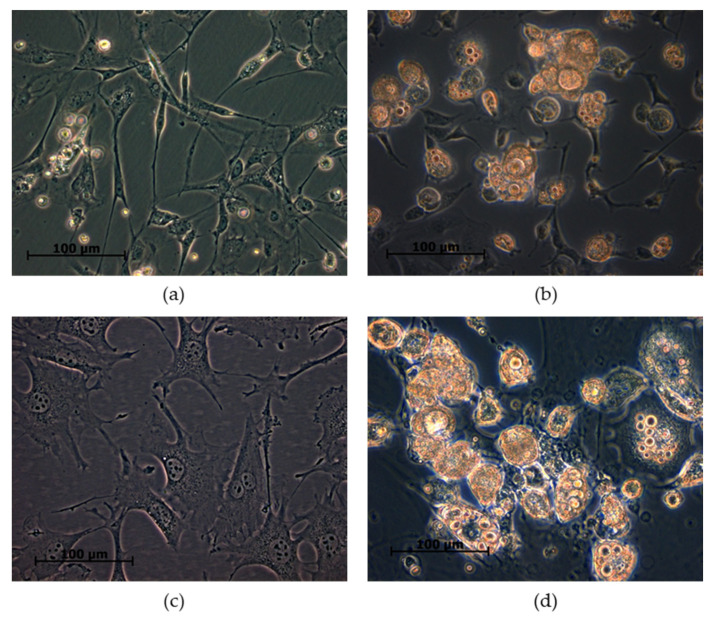
Images of rat adipocytes and 3T3-L1 cells. Undifferentiated rat preadipocytes (**a**) and adipocytes differentiated for 7 days (**b**). Undifferentiated 3T3-L1 (**c**) and 3T3-L1 cells differentiated for 7 days (**d**).

**Figure 2:**
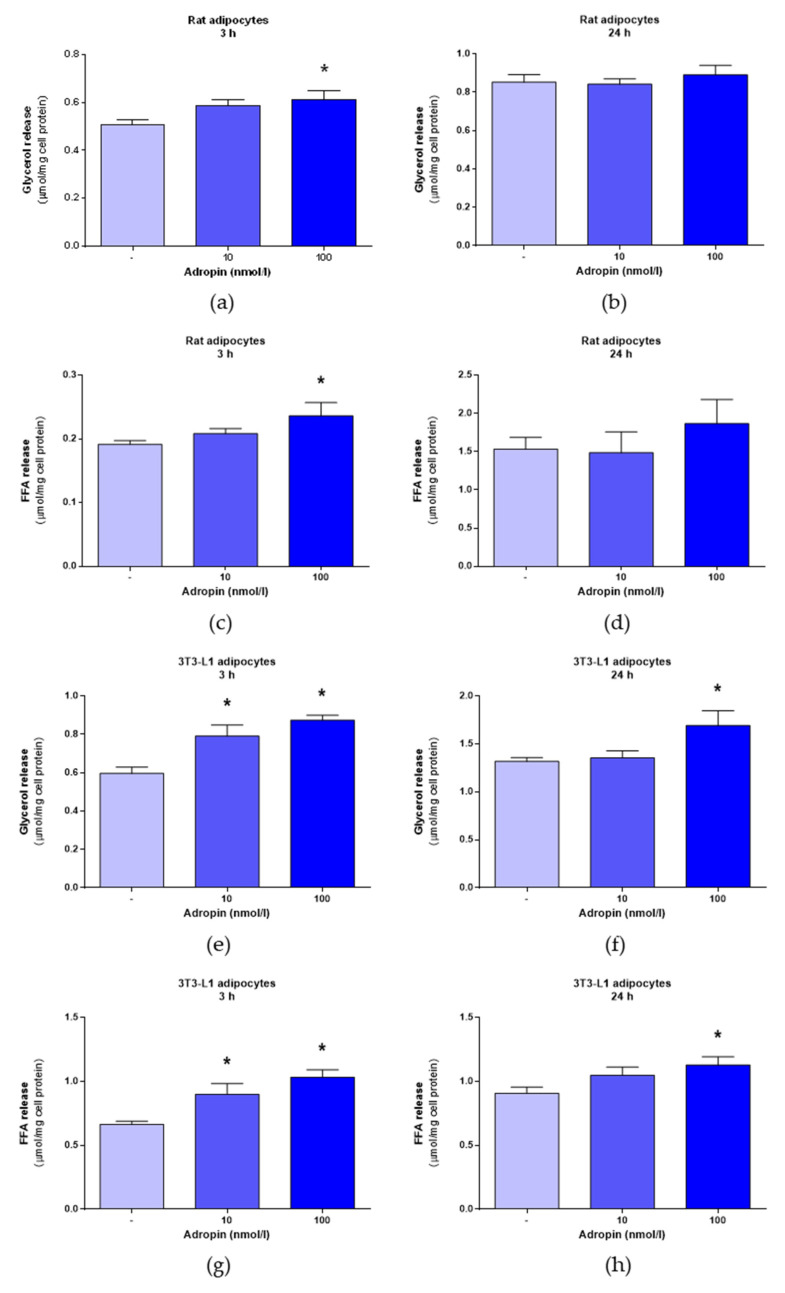
The effects of adropin (10 or 100 nmol/L) or vehicle (sterile distilled water) (-) on lipolysis in rat primary adipocytes and 3T3-L1 adipocytes. The effects of adropin (10 or 100 nmol/L) on glycerol release in rat adipocytes assessed after 3 (**a**) or 24 h (**b**) of incubation. FFA release determined in rat adipocytes exposed to adropin for 3 (**c**) and 24 h (**d**). The effects of adropin on glycerol release in 3T3-L1 cells treated with adropin for 3 (**e**) or 24 h (**f**). Secretion of FFA from 3T3-L1 incubated in the presence of adropin for 3 (**g**) or 24 h (**h**). Results are the mean ± SEM (n = 5–6). *p* < 0.05 (*).

**Figure 3:**
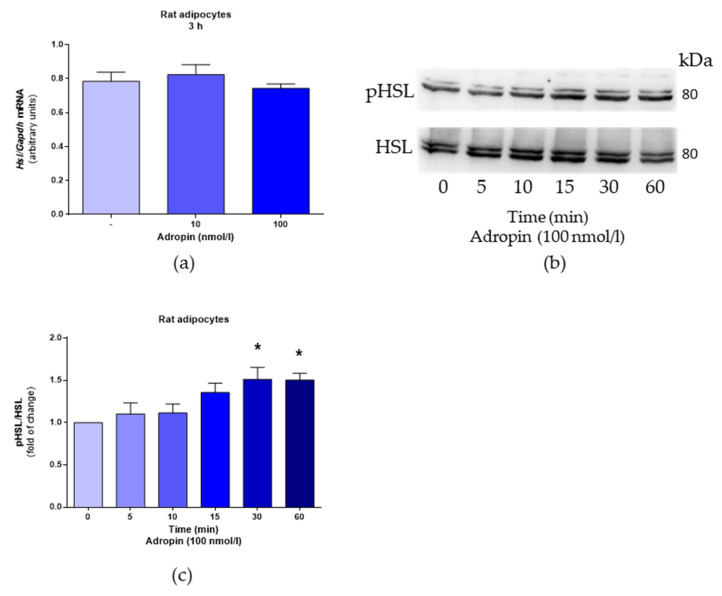
The effects of adropin on *Hsl* mRNA expression and HSL phosphorylation in rat adipocytes. *Hsl* mRNA determined in cells exposed to adropin for 3 h or vehicle (sterile distilled water) (-) (**a**). Phosphorylation of HSL in cells treated with adropin (100 nmol/L) for the indicated time points (**b**,**c**). Note the presence of two bands (84 and 89 kDa) corresponding to two HSL isoforms [21]. Results are the mean ± SEM (PCR n = 6, Western blot n = 4). *p* < 0.05 (*).

First, we evaluated the effects of adropin on lipolysis in differentiated rat primary preadipocytes. The successful differentiation of both white fat precursor cells is shown in Figure 1. It is noteworthy that undifferentiated rat white primary preadipocytes (Figure 1a) and 3T3-L1 cells (Figure 1c) display fibroblast-like morphologies and an absence of lipid droplets. By contrast, rat adipocytes (Figure 1b) and 3T3-L1 cells (Figure 1d) differentiated for 7 days have a characteristic spherical shape and contain lipid droplets. As shown in Figure 2a,c, adropin (100 nmol/L) increased glycerol and FFA release in rat adipocytes incubated for 3 h. By contrast, adropin failed to modulate glycerol and FFA release in rat adipocytes exposed to adropin for 24 h (Figure 2b,d). Furthermore, the release of glycerol (Figure 2e–f) and FFA (Figure 2g–h) increased in differentiated 3T3-L1 cells incubated with adropin (10 and/or 100 nmol/L) for 3 and 24 h. These results show that adropin stimulates lipolysis in rat primary adipocytes and 3T3-L1 cells. Nevertheless, the observed changes were moderate.

Next, we studied the effects of adropin on the mRNA expression and phosphorylation of hormone-sensitive lipase (HSL) in rat adipocytes. As shown in Figure 3a, adropin had no effect on *Hsl* mRNA expression assessed in rat adipocytes treated with adropin (10 and 100 nmol/L) for 3 h. However, adropin (100 nmol/L) promoted HSL phosphorylation in these cells (Figure 3b,c).

### 3.2. Adropin Inhibits Lipogenesis but Fails to Modulate Glucose Uptake in Rat Primary Preadipocytes

**Figure 4:**
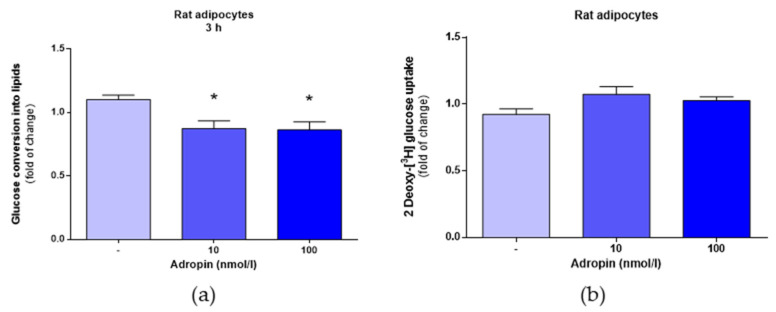
The effects of adropin on lipogenesis and glucose uptake in rat preadipocytes. Incorporation of (C14) glucose into lipids in cells exposed to adropin (10 or 100 nmol/L) or vehicle (sterile distilled water) (-) for 3 h (**a**). Glucose uptake in adipocytes treated with adropin (10 or 100 nmol/L) for 30 min (**b**). Results are the mean ± SEM (*n* = 8). *p* < 0.05 (*).

As shown in Figure 4a, adropin at the concentration of 10 or 100 nmol/L slightly reduced lipogenesis in rat primary preadipocytes. By contrast, adropin (10 and 100 nmol/L) failed to affect glucose uptake in differentiated rat adipocytes (Figure 4b).

### 3.3. Adropin Modulates Adipokine mRNA Expression

**Figure 5:**
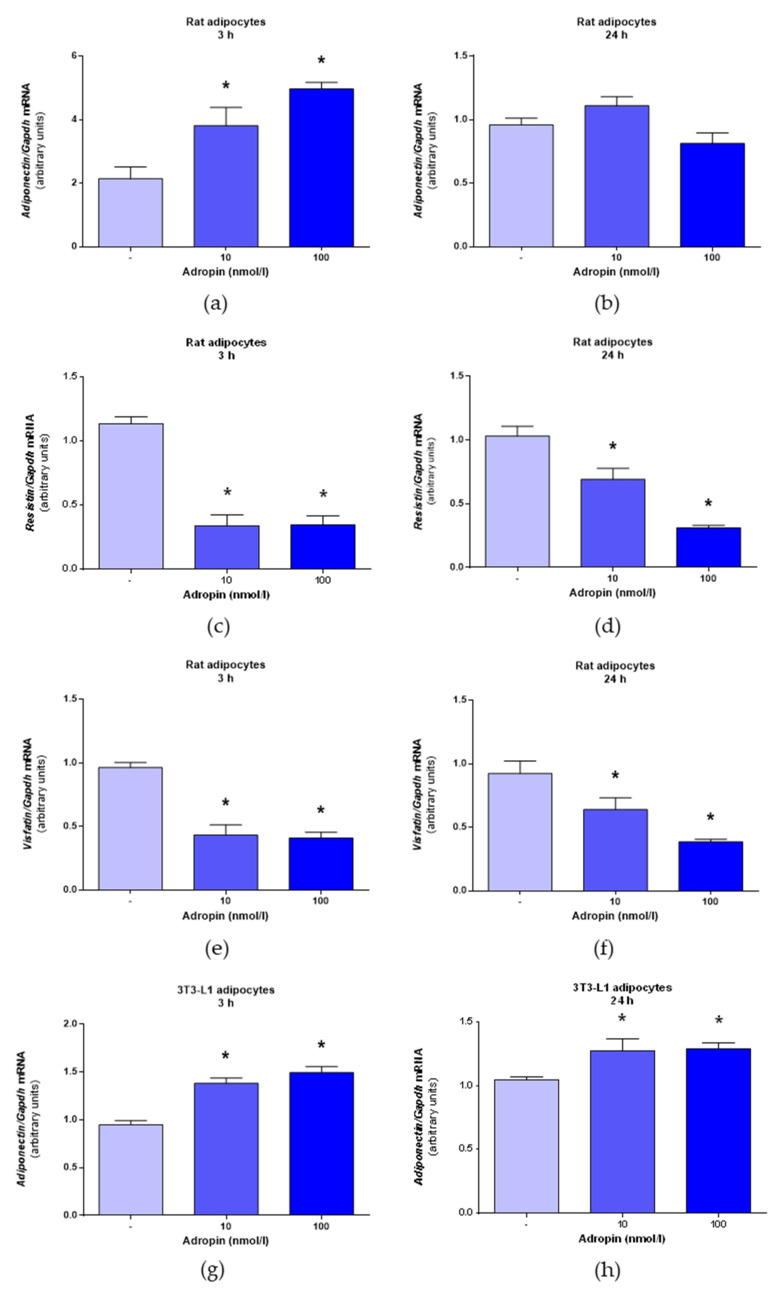

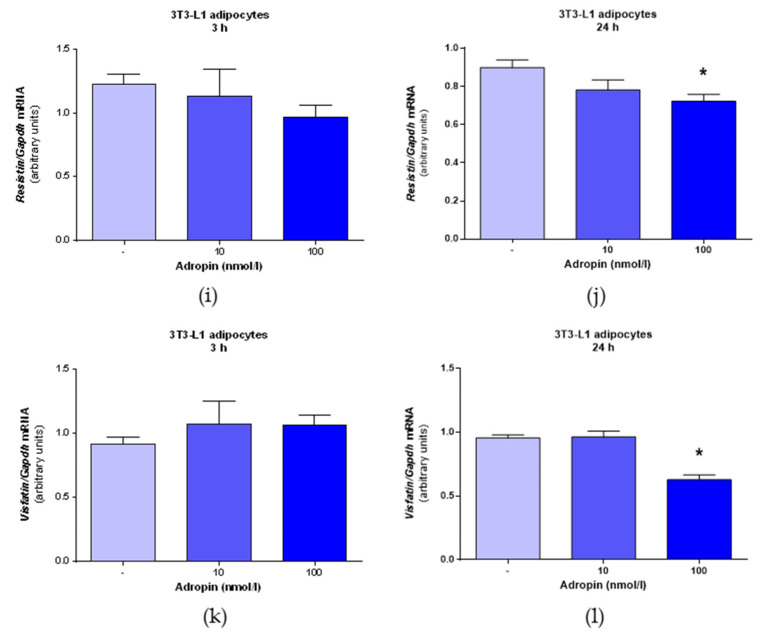
Effects of adropin on mRNA expression of adiponectin, resistin and visfatin in rat adipocytes and 3T3-L1 cells. Expression of adiponectin mRNA in rat adipocytes treated with adropin or vehicle (sterile distilled water) (-) for 3 (**a**) or 24 h (**b**). Expression of resistin mRNA in rat adipocytes treated with adropin for 3 (**c**) or 24 h (**d**). The mRNA levels of visfatin assessed in rat adipocytes incubated in the presence of adropin for 3 (**e**) or 24 h (**f**). Expression of adiponectin mRNA in 3T3-L1 cells treated with adropin for 3 (**g**) or 24 h (**h**). Resistin mRNA expression in 3T3-L1 cells treated with adropin for 3 (**i**) or 24 h (**j**). Visfatin mRNA levels in 3T3-L1 cells determined in cells exposed to adropin for 3 (**k**) or 24 h (**l**). Results are the mean ± SEM (*n* = 6). *p* < 0.05 (*).

Next, we evaluated the effects of adropin on the mRNA expression of selected adipokines, such as adiponectin, resistin and visfatin. As demonstrated in Figure 5a,b, adropin (10 and 100 nmol/L) promoted adiponectin mRNA expression in rat preadipocytes assessed after 3 h, but not after 24 h. By contrast, adropin (10 and 100 nmol/L) suppressed the mRNA expression of resistin (Figure 5c,d) and visfatin (Figure 5e,f) in rat adipocytes determined after 3 or 24 h of incubation. Furthermore, an increased expression of adiponectin mRNA was detected in 3T3-L1 exposed to adropin (10 and 100 nmol/L) for 3 or 24 h (Figure 5g,h). In 3T3-L1 cells, adropin (100 nmol/L) downregulated resistin (Figure 5i,j) and visfatin (Figure 5k,l) mRNA expression after 24 h, but not after 3 h. These results show that adropin promotes adiponectin expression but suppresses the expression of resistin and visfatin in rat adipocytes and 3T3-L1 cells.

## 4. Discussion

In this study, we report that adropin modulates lipid metabolism and the mRNA expression of adiponectin, resistin and visfatin in mature white fat rodent cells. Firstly, we found that acting on differentiated rat adipocytes and 3T3-L1 cells, adropin slightly promotes the secretion of glycerol and FFA, the hallmarks of lipolysis [22]. In our previous study, we found that adropin is able to downregulate the differentiation of white and brown rodent preadipocytes into mature fat cells [17,23]. Importantly, the suppressive effect on the differentiation of both types of preadipocytes was accompanied by lower intracellular lipid content, suggesting that adropin may be involved in controlling lipid metabolism. Consistently, other studies reported that adropin is able to reduce lipogenic gene mRNA expression, such as *Pparγ*, *Scd1* and *Fas* in white adipose tissue [1]. Therefore, these results suggest that adropin may affect intracellular lipid content by the suppression of lipid accumulation during adipogenic differentiation and by promoting the lipolytic activity of white mature adipocytes.

The regulation of lipolysis in white adipocytes is complex, and it is modulated by numerous metabolic, hormonal and environmental signals [24]. Nevertheless, there is convincing evidence that fat mobilization is strongly modulated by HSL [24,25]. Therefore, since we observed that adropin moderately promotes lipolysis in adipocytes, we assessed the effect of this peptide on the mRNA expression and phosphorylation of HSL. Our data indicate that adropin failed to affect *Hsl* mRNA levels but enhanced the phosphorylation of HSL at serine 563. It is worth noting that the phosphorylation of HSL at serine 563 is promoted by lipolysis-inducing factors [19,26,27,28]. These results provide evidence that adropin may modulate lipolysis to a slight extend by activating HSL in white adipocytes.

In addition to lipolysis, intracellular fat content in adipocytes is modulated by other processes, such as lipid synthesis termed as lipogenesis [29]. Therefore, to study the role of adropin in lipid metabolism in more detailed fashion, we evaluated the effects of adropin on glucose incorporation into lipids as a surrogate marker of lipogenesis [30]. Here, we found that by acting on white rat adipocytes, adropin slightly decreased the conversion of glucose into FFA. Thus, these results show that adropin moderately suppresses the synthesis of lipids de novo.

In adipose tissue, lipogenesis depends upon the availability of carbohydrates in circulation, as well as their uptake by adipocytes [31]. Therefore, we studied the ability of adropin to modulate glucose uptake in differentiated rat adipocytes. Adropin had no effect on glucose transport in rat adipocytes. Overall, these results collectively demonstrate that adropin may reduce intracellular lipid storage in adipocytes by promoting lipolysis and by suppressing de novo lipid synthesis. These results were moderate; however, these in vitro findings are consistent with the results of in vivo experiments, showing that adropin protects from adiposity [1,10].

Next, we evaluated the influence of adropin on the mRNA expression of selected adipokines involved in controlling energy homeostasis and metabolism, such as adiponectin, resistin and visfatin [32,33]. We found that adropin promoted the mRNA expression of adiponectin in rat adipocytes as well as in differentiated 3T3-L1 cells. In contrast, adropin downregulated the mRNA expression of resistin and visfatin in both types of adipocytes.

It is worth to note that adiponectin is able to improve insulin sensitivity [34], which resembles the therapeutic effects of adropin in animal models of diet-induced obesity [35]. Nevertheless, it remains to be investigated whether the beneficial effects of adropin on insulin sensitivity, reported in obese animals, are dependent upon adropin-modulated adiponectin expression.

By contrast, it was found that resistin impairs glucose tolerance and insulin action in rodents [36]. Furthermore, human and animal studies suggested that resistin contributes to inflammation [37,38,39]. Similarly, visfatin was found to induce inflammation and insulin resistance in hepatocytes [40]. Therefore, these two adipokines are instead considered as proinflammatory signals. Since adropin suppresses the expression of these adipokines, adropin can be considered as an anti-inflammatory factor (extensively reviewed in [41]). For instance, adropin displays anti-inflammatory properties in a liver in a rat model of hyperlipidemia [42]. A negative correlation between circulating adropin and proinflammatory signals, such as TNFα or IL-6, was reported in patients with obstructive sleep apnea [43]. Furthermore, a recent study showed that adropin is able to attenuate neuroinflammation in rats [44]. Thus, since inflammation leads to obesity-associated abnormalities [45], it is rational to speculate that the suppression of proinflammatory cytokine production by adropin may at least partially contribute to the attenuation of metabolic derangements reported in animal models of insulin resistance and obesity. Nevertheless, more in vivo experiments are needed to confirm this speculation.

Our study has several limitations. First of all, we did not elucidate the mechanism conferring the activation of HSL or lipolysis by adropin and its putative receptor GPR19. However, it was found that in tilapia hepatocytes, adropin promotes LPL expression via a cAMP/PKA-dependent mechanism [46], which was also implicated in the activation of HSL, and lipolysis in white adipocytes [47]. Furthermore, it remains to be investigated whether changes in the expression of adipokines are accompanied by changes in protein production and/or secretion in vitro or in vivo.

## 5. Conclusions

**Figure 6:**
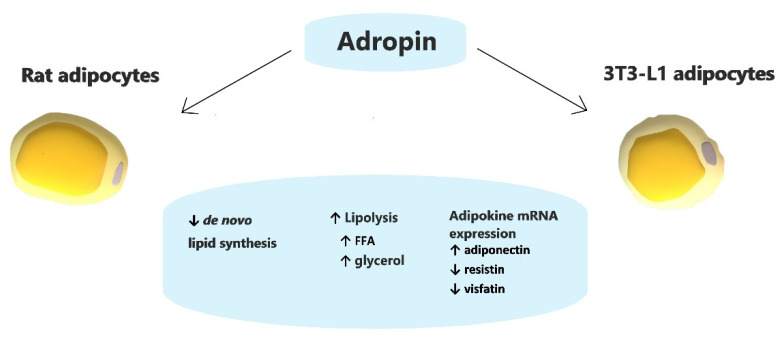
Schematic summary of the effects of adropin on white rodent adipocytes.

In summary, we found that by acting on white rodent adipocytes, adropin slightly stimulates lipolysis while suppressing de novo lipid synthesis. However, these effects were moderate. Furthermore, adropin stimulates the expression of adiponectin; however, it downregulates the expression of resistin and visfatin (Figure 6). Overall, these results show that adropin may modulate lipid metabolism and endocrine function in white adipose tissue in vitro.

## Figures and Tables

**Table 1:** Primer sequences for real-time PCR.

Gene	Left Primer (5′^>^3′)	Right Primer (5′^>^3′)	NCBI Reference Sequence
Adiponectin (rat)	tggtcacaatgggataccg	cccttaggaccaagaacacct	NM_144744.3
Gapdh (rat)	ctgcaccaccaactgcttag	tgatggcatggactgtgg	NM_017008.4
Hsl (rat)	cccaaagtaagaggcacagagt	tcctggcattcctggtctttc	NM_012859.1
Resistin (rat)	gccgctgtccagtctat	cattgctggtcagtctcc	NM_144741.1
Visfatin (rat)	cacaagagactgccggcatag	tttcccccacgctgttatgg	NM_177928.3
Adiponectin (mouse)	atctggaggtgggagaccaa	gggctatgggtagttgcagt	NM_009605.5
Gapdh (mouse)	atggtgaaggtcggtgtga	aatctccactttgccactgc	NM_001289726.1
Resistin (mouse)	tgaagatggatggcgaagtgg	gtggtgtaaatgccctgggt	NM_022984.4
Visfatin (mouse)	ccataacggcttgggggaaa	gctatcgctgaccacagaca	NM_021524.2

## Data Availability

The data presented in this study are available on reasonable request from the corresponding author.

## Abbreviations

BSA: bovine serum albumin; cAMP, cyclic adenosine monophosphate; DMEM, Dulbecco‘s Modified Eagle‘s medium; Fas, fas cell surface death receptor; FBS, fetal bovine serum; Gapdh, glyceraldehyde 3-phosphate dehydrogenase; IBMX, 3-isobutyl-1-methylxanthin; IL-6, interleukin 6; LPL, lipoprotein lipase; PBS, phosphate-buffered saline; PKA, protein kinase A; Pparγ, peroxisome proliferator-activated receptor γ; Scd1, stearoyl-CoA desaturase; SDS, sodium dodecyl sulfate; T3, triiodothyronine; TNF-α, tumor necrosis factor α.
